# Factors influencing breastfeeding continuation and formula feeding beyond six months in rural and urban households in Indonesia: a qualitative investigation

**DOI:** 10.1186/s13006-023-00586-w

**Published:** 2023-08-31

**Authors:** Bunga Astria Paramashanti, Michael J Dibley, Tanvir M Huda, Yayi Suryo Prabandari, Neeloy Ashraful Alam

**Affiliations:** 1 Department of Nutrition, Faculty of Health Sciences, Universitas Alma Ata, Yogyakarta, Indonesia; 2 Sydney School of Public Health, Faculty of Medicine and Health, The University of Sydney, Sydney, New South Wales Australia; 3 Department of Health Behavior, Environment, and Social Medicine, Faculty of Medicine, Public Health, and Nursing, Universitas Gadjah Mada, Yogyakarta, Indonesia

**Keywords:** Continued breastfeeding, Breastfeeding, Duration, Formula, Factors, Rural, Urban

## Abstract

**Background:**

Global and Indonesian guidelines suggest that breastfeeding should continue for at least the first two years of life. While many studies have focused on six-month exclusive breastfeeding practices, little is known about why mothers do not sustain breastfeeding beyond this period. This qualitative study aimed to explore factors influencing breastfeeding continuation and formula feeding beyond six months, regardless of any additional food consumed, focusing on Indonesia’s rural and urban areas.

**Methods:**

We collected the data through 46 in-depth interviews in Pati District and Surakarta City, Central Java, Indonesia. Participants were mothers, grandmothers, health care practitioners, and village *kader* (frontline female health workers). We used thematic analysis combining deductive and inductive techniques for analysing the data.

**Results:**

Rural mothers practised breastfeeding and intended to breastfeed for a longer duration than urban mothers. Maternal attitude towards breastfeeding, breastfeeding knowledge, previous experiences, and other breastfeeding strategies (e.g., enhancing maternal dietary quality) positively influenced breastfeeding sustainability. In the urban setting, mothers encountered several breastfeeding barriers, such as perceived breast milk insufficiency and child hunger and satiety, child biting, and breastfeeding refusal, causing them to provide formula milk as a breast milk substitute or supplement. In addition, families, communities, health practitioners, and employment influenced maternal decisions in breastfeeding continuation and formula-feeding practices.

**Conclusions:**

Optimal breastfeeding practices up to two years of age are determined by the individual and setting (i.e., community, healthcare, employment) factors. Providing breastfeeding education covering practical breastfeeding guidance will encourage mothers to breastfeed for longer. Such interventions should involve families, communities, health workers, and the work environment as a breastfeeding support system. Policymakers should develop, enforce, and monitor the implementation of breastfeeding policies to protect, promote, and support breastfeeding in households, communities, health systems, and work settings.

## Background

Breastfeeding benefits children, mothers, and society. It contributes to a healthier, more educated, equitable, and environmentally sustainable world [1]. By extending the breastfeeding duration, children would have a reduced risk of morbidity and improved intelligence than those with shorter time or without breastfeeding [1, 2]. In addition, even a slight increase in breastfeeding coverage and period could give significant economic savings [3].

The World Health Organization (WHO) and United Nations Children’s Fund (UNICEF) have recommended mothers exclusively breastfeed their infants for the first six months of age and then continue breastfeeding for up to two years or above [4]. Global Breastfeeding Collective has set 2030 breastfeeding targets that include 1-year and 2-year continued breastfeeding at 80% and 60%, respectively, to increase the commitment to breastfeeding’s promotion, protection, and support [5]. Despite the global recommendations and the importance of increasing breastfeeding duration, there is no national target for breastfeeding continuation beyond the exclusivity period in Indonesia. Based on the most current Indonesia Demographic and Health Survey in 2012 and 2017, the proportion of continued breastfeeding in years one and two has stagnated at 77% and 55%, respectively. Furthermore, breastfeeding’s median duration had decreased from 23.9 months to 21.8 months over the last two decades [6, 7].

In contrast to exclusive breastfeeding which was often associated with maternal breastfeeding knowledge, delivery mode, antenatal and postnatal counselling, and maternal occupation, a systematic review study revealed that socioeconomic and demographic factors greatly influenced breastfeeding maintenance for at least 12 months. These factors included maternal age, maternal education, family income, and living residency [8]. In low- and middle-income countries, breastfeeding is less popular among higher-income, better-educated, and urban women. It is often perceived as inferior and unsophisticated. These women would prefer breastmilk substitutes regarded as modern and prestigious [1]. Even urbanicity was associated with breastfeeding for less than two years in India. Women who lived in urbanised villages tended to have improved education, income, and household wealth, suggesting that they had greater affordability and social desirability of commercial breastmilk substitutes [9]. Two nationwide Indonesian studies found inconsistent results of breastfeeding duration with household socioeconomic status and living residency among children under two years [10, 11]. Since breastfeeding continuation depends on contextual factors and maternal sociodemographic characteristics, it is essential to understand the underlying factors behind breastfeeding maintenance in different sociocultural contexts [8].

A complete understanding of breastfeeding influencing factors is essential to develop breastfeeding promotion programs that help improve breastfeeding duration. Many studies have explored the factors behind maternal breastfeeding decisions in low- and middle-income countries. However, most of them only focused on exclusive breastfeeding during the first six months of life [12–15]. Besides, these studies involved a single type of participants [12, 13, 15] and study settings [13–15], making it challenging to explain breastfeeding practices across different points of view and contexts. Additionally, breastfeeding up to two years of life or beyond is critical but often understudied. Less is known about why mothers in developing countries stopped breastfeeding altogether during this period. Thus, this study aimed to explore factors influencing breastfeeding continuation and formula feeding beyond six months, regardless of additional food in rural and urban areas of Central Java Province, Indonesia.

## Methods

### Study design and setting

We conducted a qualitative study through in-depth and key informant interviews to explore breastfeeding continuation until 23 months old regardless of additional food and its influencing factors among rural and urban mothers. This study took place in one rural village in Pati District and two urban villages in Surakarta District, Central Java Province, Indonesia, from January to April 2020.

Known as the “heart” of Javanese culture, Central Java is one of 34 Indonesia’s provinces between West Java and East Java Province on Java Island. The population mainly was the Javanese ethnic group and Moslems [16]. In terms of exclusive breastfeeding, Central Java was among the provinces with the lowest rates (< 37%) [17]. We selected Pati District and Surakarta City as our study locations because the two areas had complex child malnutrition issues. For example, Pati District had the highest wasting prevalence (14%) and second-highest stunting prevalence (46%) in Central Java, while Surakarta City had the highest overweight (18%) and stunting (57%) prevalence [18].

### Participants

Our participants were mothers of young children aged 6–23 months, stratified by rural or urban settings. We purposively recruited mothers based on the growth monitoring lists from *posyandu*, an integrated health service post at the village level. After explaining our study, a local midwife in Pati District and a local nutritionist in Surakarta City led the field research team to village *kader* before reaching participants. *Kader* are local frontline female health workers who live within the community they serve. They assist the village midwife in organising *posyandu*, including providing health information and mobilising the community to participate *posyandu* activities. *Kader* would pay a home visit if mothers did not join *posyandu* or if the children were undernourished [19]. Both *kader* and the midwife knew mothers or grandmothers in this study since they were listed as *posyandu* participants. Most mothers or grandmothers in this study knew each other since they lived in the same community and met during *posyandu* activities.

**Fig. 1:**
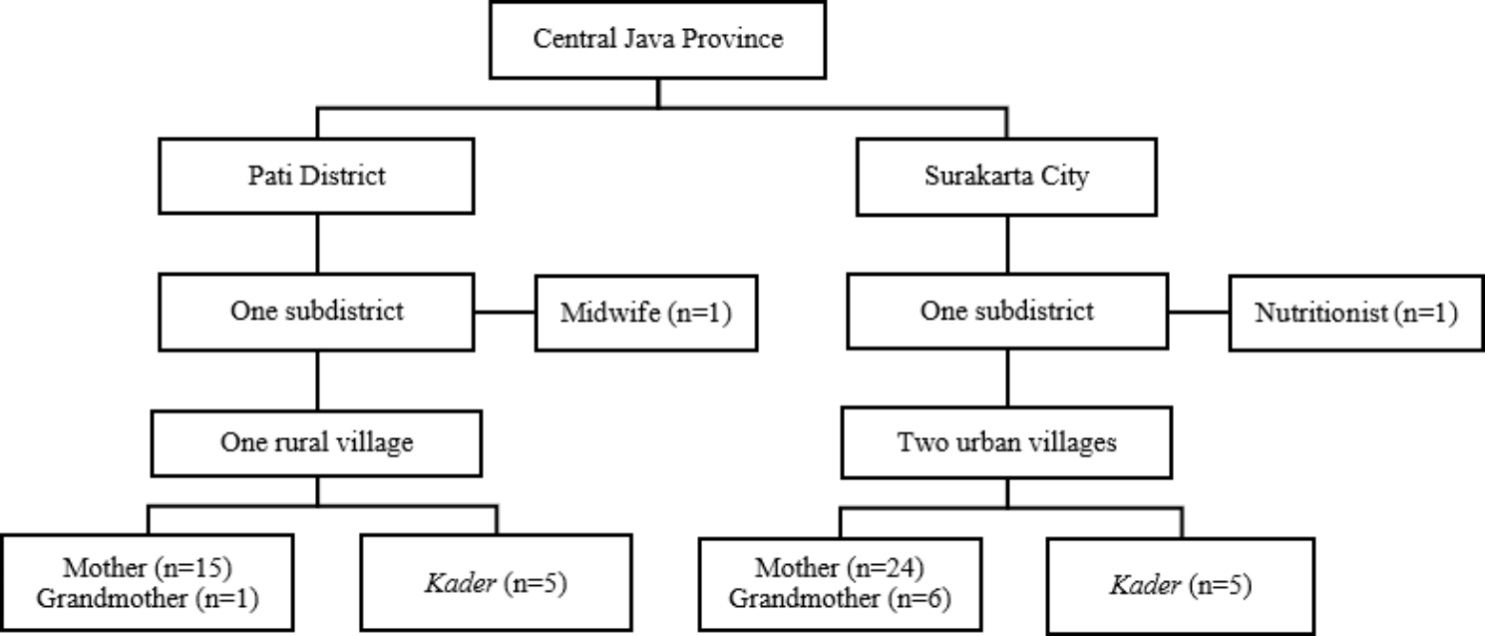
Sampling frame of the study

We interviewed both mothers and grandmothers of children whose grandmothers cared for them while their mothers worked. We interviewed a nutritionist, a midwife, and ten village *kader* to investigate their experience with the community related to breastfeeding practices. Face-to-face interviews were conducted with caregivers either at the *posyandu* site or at their house, depending on their preference. We separately interviewed key informants face-to-face in their working areas (i.e., *posyandu* or primary health centre) or via phone. Figure 1 depicts the sampling frame for this research.

### Data collection

We developed interview guidelines in English and then translated them into Bahasa Indonesia. The questions covered breastfeeding continuation up to 23 months and related facilitating and inhibiting factors. The lead author (BAP) and three research assistants with qualitative research experience conducted in-depth interviews with primary caregivers in rural (n = 16) and urban (n = 30) settings. Before the interview, we explained our study to all participants and requested their consent to participate in this study. Firstly, we asked about their sociodemographic data and filled in a brief structured questionnaire. Afterwards, we interviewed them with open questions based on guidelines and probed to obtain more detail when necessary. We audio-recorded each interview, which lasted around 30–45 min, and took notes of key points to support data capturing.

### Data analysis

We used deductive-inductive thematic analysis to understand the factors behind participants’ breastfeeding continuation [20, 21]. First, we grouped these factors into facilitators and barriers to sustaining breastfeeding, then, we classified them based on the level of determinants proposed by Rollins et al. [1, 22]. We also used this framework to group formula feeding factors based on the different level factors [1, 22], while adopting the specific framework of commercial feeding for discussion [23]. Additionally, we developed relevant themes based on the data and shared them with field research assistants to verify these data aligned with the live interview experience and notes.

Using the iterative process, the six-phase process by Braun and Clarke [20, 21] helped facilitate data analysis in this study. First, following verbatim transcription and translation of audio recordings into English, BAP read the transcripts repeatedly to facilitate familiarisation and immersion. Second, BAP produced a list of priori codes using the interview guides. The initial round of coding was made based on the mothers’ and grandmothers’ interviews, followed by *kader* and health workers’ interviews to compare and confirm mothers’ and grandmothers’ responses. BAP then discussed the coding with NAA to modify the list and develop a draft codebook. Subsequently, BAP continued the coding, including any necessary code rose from the interview, and provided relevant quotes. Third, BAP grouped these codes into categories in a matrix created using a spreadsheet to compare breastfeeding continuation practices in rural and urban settings, which later constructed initial candidate themes. Fourth, BAP and NAA reviewed potential themes until reaching a significant agreement. Any disagreement was resolved through exhaustive discussion. Afterwards, BAP discussed and finalised the themes and sub-themes related to the research questions and dataset with NAA.

To ensure the credibility of our analysis, we conducted triangulation and persistent data observation [24]. Data source triangulation was made based on different perspectives from mothers, grandmothers, *kader*, a nutritionist, and a midwife. Investigator triangulation was applied by involving all field research teams in both data collection and transcription processes. To minimise the interpretative bias, BAP analysed the data with a co-author and experienced qualitative researcher (NAA) who was absent from the data collection process.

## Results

We conducted a total of 46 in-depth interviews with caregivers of children aged 6–23 months. Table 1 presents the characteristics of mother-child pairs in rural and urban Central Java, Indonesia. The mean age of the rural and urban mothers was 29.7 and 29.4 years, respectively. Most rural mothers completed junior high school (87.4%), while half of the urban mothers completed senior high school (50.0%). Around six per cent of rural mothers worked outside the house, whereas one-third of urban mothers were employed. Approximately 56% of children in both settings were female, with the mean age of children being 13 months in rural areas and 14 months in urban areas.

**Table 1:** Characteristics of mothers and children aged 6–23 months in rural and urban areas of Central Java, Indonesia

Characteristics	Rural (n = 16)	Urban (n = 30)
	mean ± SD or %	mean ± SD or %
Maternal age (in years)	29.7 ± 5.9	29.4 ± 6.7
Maternal education		
Completed junior high school	87.4	33.3
Completed senior high school	6.3	50.0
Completed university	6.3	16.7
Maternal occupation		
Not working/housewife	93.7	70.0
Working outside the house	6.3	30.0
Child age (in months)	13.0 ± 4.5	14.0 ± 5.3
Child sex		
Girls	56.2	56.7
Boys	43.8	43.3
Breastfeeding duration at the time of the interview (in months)	13.0 ± 4.5	9.7 ± 5.7

The main themes that emerged in this study included facilitators and barriers to sustaining breastfeeding beyond six months and factors of formula feeding. Table 2 describes the themes, sub-themes, and illustrative quotes of our study participants.

**Table 2:** Themes and sub-themes related to breastfeeding continuation at the individual and setting levels in rural and urban Central Java, Indonesia

Level of determinants	Theme 1: Facilitators of breastfeeding continuation		Theme 2: Barriers to breastfeeding continuation		Theme 3: Drivers of formula feeding	
	Rural	Urban	Rural	Urban	Rural	Urban
Individual	Attitudes toward breastfeeding	Attitudes toward breastfeeding		Perceptions of inadequate milk supply	Perceptions of inadequate milk supply	Perceptions of inadequate milk supply
	Knowledge of general health and economic benefits of breastfeeding	Knowledge of health benefits of breastfeeding		Perceptions of child hunger	Perceived formula feeding benefits	Perceived formula feeding benefits
	Breastfeeding experience with a previous child	Breastfeeding experience with a previous child		Child biting		
	Problem-solving strategies	Problem-solving strategies		Child refusal		
Settings	Breastfeeding education from a highly respected midwife	Breastfeeding education from various resources	Community views on breastfeeding up to two years	Community views on breastfeeding up to two years	Maternal employment	Maternal employment
			Maternal employment	Maternal employment		

### Theme 1: facilitators of breastfeeding continuation

All rural mothers still practised breastfeeding on the interview date. In the urban setting, around half of the mothers maintained breastfeeding (n = 16). Among rural and urban mothers who continued breastfeeding, we identified several factors that positively influenced its sustainability, such as breastfeeding attitude, breastfeeding knowledge, previous breastfeeding experience, and problem-solving strategies to extend breastfeeding duration.

#### Attitudes toward breastfeeding

Generally, mothers regarded breast milk as the best nourishment for infants and young children. They often compared its superiority over formula milk. For example, a mother said, *“Breastmilk is the best (food) for a child.”* (an urban mother aged 38 years). Similarly, an urban grandmother said, *“Breastmilk is the number one priority for babies”* (an urban grandmother). Some rural mothers also viewed breastfeeding as the child’s right to provide for at least two years as stated by a mother, “*Because breastfeeding is an obligation of a mother. It is the child’s right.”* (a rural mother aged 26 years).

#### Breastfeeding knowledge

Most mothers were well-informed about breastfeeding benefits for their children. They agreed that breastmilk could prevent infections and optimise growth and brain development in both settings. A rural mother summarised, *“To be healthy, not getting sick easily…”* (a rural mother aged 21 years). Nonetheless, we also found that some rural mothers were unsure about other breastfeeding advantages by stating *“I don’t understand”, “What else?”, “What is it?”, “Is that correct?”*. Meanwhile, urban mothers generally be more specific when describing breastfeeding benefits. For example, *“Breastmilk can improve her immune system and for her cognitive development.“* (an urban mother aged 31 years).

We noted that rural mothers considered an economic benefit of breastfeeding that we did not find in the urban setting. For example, a mother said, *“I breastfeed my child so that she can grow healthy and normal… and reduce our (financial) burden. It (the information) is from the midwife and the primary health centre’s staff. I think the advice was correct.”* (a rural mother aged 37 years).

Most urban mothers and some rural mothers believed that breastfeeding should continue for two years. For example, a mother said, *“(I will breastfeed him) Maybe for two years. At two years, breastfeeding will be stopped.”* (an urban mother aged 20 years). However, breastfeeding should last as long as the child wants it for a few rural mothers. For example, a mother explained, *“Just like her sister (who was breastfed until three years), I will breastfeed her as long as she wants… I will not force weaning her.”* (a rural mother aged 31 years).

#### Previous breastfeeding experience

Rural and urban mothers reflected on the positive breastfeeding experience of them or their peers, making them feel confident about sustaining breastfeeding. A mother said, *“Just like my first child. I produced breastmilk immediately after birth. Now he is still breastfed. No formula, but breast milk. (I will breastfeed him) Maybe for two years. At two years, breastfeeding will be stopped. His brother was (breastfed) for more than two years.”* (an urban mother aged 20 years). Yet a rural mother who planned to breastfeed for more than two years described her experience with her first child, *“I weaned her sister after three years. After she reached 2.5 years, I breastfed her rarely.”* (a rural mother aged 31 years).

Mothers might learn not only breastfeeding practices based on positive experiences but also negative ones. Urban mothers’ regretful feelings in the past encouraged them not to repeat such experiences. A mother said, *“It was my first child that I gave him formula milk… When I was thinking about him, I felt pity for him. For my second child, she is breastfed.”* (an urban mother aged 26 years).

#### Problem-solving strategies to continue breastfeeding

Both rural and urban mothers who sustained breastfeeding took several actions when faced with breastfeeding challenges. For instance, some urban and rural mothers consumed green vegetables and fish, increased their meal portion size, and used galactagogues to boost milk production. One mother said, *“I took the capsule and herbal drink because the milk production was not smooth… I meant I could not produce much.”* (an urban mother aged 24 years).

Another effort used by mothers was to quit their job. One rural and one urban mother decided to leave their work for breastfeeding for up to two years. A rural mother who used to be a labourer described how her husband’s persuasion convinced her to decide: *“When I was still working, my husband encouraged me to quit. I should only work after my daughter aged two years old.”* (a rural mother aged 27 years).

#### Breastfeeding education

Both rural and urban mothers mostly received breastfeeding education from health providers. In rural, the village midwife was a highly respected health professional working who delivered infant and young child nutrition, including breastfeeding. A mother explained her adherence to breastfeeding recommendations: *“I followed the suggestion from the midwife. It is better to provide breastmilk than formula milk. It is healthier.”* (a rural mother aged 39 years).

A rural midwife explained about community education groups held in her working areas:

*“Mothers practised appropriate breastfeeding because they had joined Pregnant Women and Toddler Classes. During these classes, there was information about breastfeeding, so they knew about breastfeeding importance. (I told them that) Breast milk is more convenient, cheaper, and more hygienic than formula.”* (a rural midwife).

In urban areas, we found more varied resources for breastfeeding education, including nutritionists, local midwives, general practitioners, and university students who conducted community services.

*“I received breastfeeding information since I was pregnant. It was during my antenatal visit to the primary health centre. There is also information from Posyandu. In Posyandu, the educators are kader and the midwife.”* (an urban mother aged 27 years).

*“Health staff from the primary health centre gave routine education on child nutrition. That included breastfeeding. There were also many university students who gave education on breastfeeding.“* (an urban kader).

### Theme 2: barriers to breastfeeding continuation

All of our rural participants continued to breastfeed their children until the interview date. In contrast, nearly half of the urban mothers had stopped breastfeeding their young children, which mainly occurred below twelve months. There was a range of explanations for breastfeeding discontinuation among urban mothers. At the maternal factors, these barriers included perceptions of inadequate milk supply and perceptions of child hunger. Child factors were child biting and breastmilk rejection. Maternal employment also influenced mothers to terminate breastfeeding.

Since all rural mothers were breastfeeding until the interview date, the following are barriers to sustaining breastfeeding among urban mothers:

#### Perceptions of inadequate milk supply and child hunger

The most common reason for breastfeeding cessation in urban settings was maternal perception of milk supply. Urban mothers interpreted child fussiness and high appetite as hunger cues because breastmilk could no longer satisfy their children.

*“My breastmilk was decreased a lot. So, it is like she was always fussy when she drank less.”* (an urban mother aged 30 years).

*“…(My) breast milk was not sufficient. He (my son) kept crying. When I pumped, I was very stressed. At first, I gave him both breastmilk and formula because he drank a lot. Well, now only formula milk since… twelve months.”* (an urban mother aged 31 years).

*“Many mothers also complained about breast pain… and that their breast milk did not come out anymore.”* (an urban kader).

#### Child biting and rejection

A few urban mothers reported that they stopped breastfeeding because they could not endure the pain caused by child biting, making their nipples sore and cracked. For example, a mother explained, *“He has so many teeth already. He suckled until my nipples ripped. It hurt, so I didn’t breastfeed him. At the same time, I weaned him.”* (an urban mother aged 24 years).

Many mothers also mentioned breast milk refusal by their children. While some were unsure about the underlying factor, some perceived that breast milk rejection might be caused by low milk production, child hunger, illness, and bottle-feeding preference. They narrated:

*“She refused… refused by herself. She did not like direct breastfeeding but asked for a bottle (feeding). I produced breastmilk little by little. I only produced from the left side, and the right side was not coming out. Later, the breast milk did not come out at all.”* (an urban mother aged 23 years).

#### Community views on breastfeeding up to two years

Following the previous national recommendation, it was a general knowledge among rural and urban communities and health workers that breastfeeding should be up to two years, not beyond. This view might lead to reduced breastfeeding duration for two years only. A mother said, *“I know that breastfeeding should be up to two years.“* (an urban mother aged 29 years). However, since our participants were mothers with children aged 6–23 months, we could not explore breastfeeding continuation beyond this period.

During the data collection, health workers still delivered breastfeeding for up to two years messages to the community.

*“For infants above six months, we always encourage mothers to continue breastfeeding them until two years.“* (an urban nutritionist).

*“Previously, many mothers provided breast milk until their children grew up, such as the above two years… even until their children entered a kindergarten school. But new young mothers were already educated. We educated them during Toddler and Pregnant Women Class, so they do not practice it now. They only offered breast milk for two years.”* (a rural midwife).

#### Maternal employment

One-third of urban mothers were employed. Some supplemented breastfeeding with formula feeding, while others gave up breastfeeding altogether. Few working mothers expressed and stored breastmilk, but only one succeeded in breastfeeding continuation up to the interview date. A mother who eventually discontinued breastfeeding narrated:

*“I used to store my breast milk in the freezer. Then, I could not because it was hard to squeeze (breast milk hand expression). If I squeezed, it (breast milk) was only a little. I had the pumping tool, Maam… but the milk did not come out. But if she suckled, the milk supply became a lot. I also ate a lot, not a little. Maybe it is because I breastfed her every day in the past. Now I do not breastfeed her during the day.”* (an urban mother aged 28 years).

*“The inhibiting factor was maternal employment because they left their children at home while they were working, although some mothers pumped their breasts.”* (an urban *kader*).

Conversely, most rural mothers were housewives. Some of them related being with their children at home make breastfeeding possible. For example, a mother said, *“I do not work outside, so I do not give formula milk, but breast milk.”* (a rural mother aged 31 years). However, we did not ask specifically how the workload outside and inside the home might affect the mother’s flexibility in breastfeeding.

### Theme 3: factors influencing formula feeding

We found that formula feeding in rural areas was not as common as in urban areas. Even though few rural mothers had provided formula milk during early infancy before their breast milk came out or due to low breastmilk supply, they quit it because their milk supply was abundant, formula milk rejection by the children, or not suggested by health workers.

*“I had bought formula, but I did not use it. He (my son) did not like it, and if I forced him, he would vomit.”* (a rural mother aged 36 years).

*“My husband advised me to give formula milk so that her (my daughter’s) body weight increased. But, when she was sick and we brought her to the clinic, the doctor said that her body weight was fine. So, I didn’t give her formula (anymore).“* (a rural mother aged 29 years).

*“I followed the suggestion from the midwife. It is better to provide breastmilk than formula milk. It is healthier.”* (a rural mother aged 39 years).

We did not ask specifically about formula milk advertisements. However, the rural midwife described that the newest regulation did not allow health workers to promote breastmilk substitutes. On some occasions (e.g., when the children were severely malnourished), the midwife and village *kader* would visit mothers at their houses to check their feeding practices. The midwife said that some mothers would hide the formula milk when they visited. These findings suggested that rural health workers did not encourage formula milk and that rural mothers knew formula feeding was not advisable.

Only one rural mother offered formula milk as breastmilk supplementation due to the perceived low milk supply and working outside the house. Conversely, formula feeding was a widespread practice among urban mothers. While few urban mothers had started to introduce bottle feeding before six months, more mothers provided formula milk as the child grew older. For all mothers who provided their children with formula milk, we asked the reasons behind such practices. The influencing factors were perceptions of low breast milk supply, perceived formula milk benefits, and maternal employment.

#### Perceptions of inadequate milk supply

Maternal perception of breast milk insufficiency was the most reported reason to offer formula feeding, either to supplement or substitute breast milk. Some of these mothers had started to offer formula milk before six months.

*“My breastmilk is only a little, so I also give him formula. Previously, I told my mother that my breastmilk is lacking and asked her opinion about formula feeding. She just agreed.“ (a rural mother aged 26 years)*.

*“She was not satisfied because my breastmilk didn’t come out. (She) Always cried… then I gave her formula.” (an urban mother aged 23 years)*.

*“As far as I know, some mothers already provided their babies with formula milk before six months… it’s because they thought their breast milk is inadequate, then their babies are less satisfied.“ (a rural kader).*

*“I am sure that all of the mothers here wanted to breastfeed their babies fully. Some mothers had issues like their breastmilk did not come out at all. But, mostly… the problem was low breastmilk supply. So, they gave formula milk to their babies.“ (an urban kader)*.

#### Perceived formula milk benefits

Some urban mothers offered formula feeding to their children because they thought formula milk had several benefits linked to their children’s growth and development. For some mothers, they decided to give formula milk by themselves, while others were supported by their husbands or mother.

*“My husband advised me to give formula milk so that her (my daughter’s) body weight increased. But, when she was sick and we brought her to the clinic, the doctor said that her body weight was fine. So, I didn’t give her formula (anymore).“ (a rural mother aged 29 years)*.

*“If I only gave her breast milk, I would feel pity for her. Her body weight often did not increase but kept decreasing. So, I gave her the formula to not look too weak like that. Her development might also be slowed (if she only consumed breast milk).” (an urban mother aged 47 years)*.

*“His mother gave him formula milk when he was three months old. I just followed her. It was when his body weight did not increase, so she gave her formula milk. Then, his body weight increased 100 grams… little by little.“ (an urban grandmother).*

#### Maternal employment

All urban mothers who discontinued breastfeeding altogether provided their children with formula milk. Most mothers who still maintained breastfeeding also offered formula feeding as a supplement. They managed the feeding schedule based on their daily routine.

*“My son consumes formula milk, especially when I am working outside.“ (a rural mother aged 26 years).*

*“When her mother works, like today… her mother has morning shift, so he is with me… I give him formula milk. He will get breastmilk when his mother comes home.“ (a rural grandmother).*

*“I breastfed him since he was born, but now I also give him formula milk because I left him for work. It (Formula feeding) was started when he was four months. I tried to store my milk, but I felt pity for his grandmother. His grandmother was already busy with (managing) her retail shop, while at the same time caring for my child… But, when I am home, I fully breastfeed him.“ (an urban mother aged 27 years)*.

*“Now still breastmilk. At day, she had formula milk… at night, she had breastmilk.”* (an urban grandmother).

## Discussion

### Main findings

Our study compared breastfeeding continuation practices regardless of additional food among rural and urban mothers in Central Java Province, Indonesia. We found that rural mothers were more successful in sustaining breastfeeding for a longer duration than urban mothers. Most mothers who were still breastfeeding intended to breastfeed until their children reached two years of age, except for some rural mothers who decided to breastfeed for around three years. Breastfeeding enabling factors included maternal attitude towards breastfeeding, breastfeeding knowledge, previous experiences, problem-solving strategies to continue breastfeeding (i.e., improving dietary quality, using galactagogues, quitting a job), and breastfeeding education. Although these facilitators were familiar with both areas, urban mothers faced several barriers (i.e., perceptions of low breast milk supply, employment, child biting and refusal), causing them to stop breastfeeding. Community views on recommended breastfeeding duration limited breastfeeding continuation beyond two years of age in both areas. Most urban mothers provided formula milk either as a breastmilk substitute or a supplement. Perceived breast milk insufficiency was the leading cause of both breastfeeding termination and formula feeding. In addition, perceptions of formula milk benefits and women’s employment were drivers of formula feeding practices. Following Rollin et al.’s conceptual model [1, 22], we discussed the determinants of breastfeeding continuation up to two years of age and grouped them according to individual and setting-level factors that emerged from this study. We also related these factors with structural determinants.

### Determinants of sustaining breastfeeding beyond six months

#### Individual factors

At the individual level, maternal attitudes and knowledge of breastfeeding led to the continuation of breastfeeding in rural and urban settings. In line with previous studies [25–28], most mothers in this study were breastfeeding because they believed breastmilk was the perfect food for their children and every child’s right. Many of them viewed breastfeeding as healthier than formula feeding, which we found in earlier research [27, 29], while rural mothers notably considered breastmilk affordable. They chose breastmilk because it is freely available instead of giving formula milk that they felt more burdensome, similar to previous studies in Pakistan [30] and Tanzania [12]. Thus, breastfeeding education strategies should target mothers during the antenatal and postpartum periods to improve maternal attitudes toward promising breastfeeding practices. While recognizing breastfeeding benefits is important, incorporating breastfeeding education and discussion on how to cope with breastfeeding issues may result in optimal breastfeeding practices.

Maternal perceptions toward breastfeeding influenced maternal decisions on whether to continue or terminate breastfeeding. Between six months and two years postpartum, perceived insufficient milk (PIM) was a critical factor that led urban mothers to discontinue breastfeeding altogether, provide formula feeding, or both, which we did not find in rural settings. In common with previous qualitative research, PIM was typical among urban mothers in other countries, such as India [31], China [32], and Thailand [33]. While breast milk supply can be estimated objectively [34], it is also subjective to mothers’ beliefs [35]. Urban mothers often related PIM to other breastfeeding misconceptions, such as child satiety (e.g., cranky child), breast milk rejection (e.g., the child refused to suckle), and impaired growth and development (e.g., weak appearance). As in our study, other research has also found that mothers may mistake unsettled infant behaviour (e.g., fussiness, wakefulness, tiredness) for hunger signs [22, 36–38]. These concerns are used by formula milk industries to advertise their products [22, 39]. Our urban mothers provided the formula to complement or substitute breast milk due to PIM, as shown in earlier publications [22, 25, 31]. To eliminate factors that may lead to PIM, health workers should inform mothers about typical baby behaviour as part of the human development stage [22, 37], appropriate hunger and satiety cues [22, 37], responsive feeding practices [40], nutrition for lactating mothers [38, 41], and latching and positioning [41], and continuous breastfeeding support [41–43]. Besides, it is necessary to help mothers recognise whether their milk supply is insufficient by assessing the frequency and amount of breastfeeding, the number of wet diapers, and the child’s body weight.

Only a few urban mothers who could overcome breastfeeding hurdles sustained breastfeeding without formula. They were mothers who learned from their breastfeeding experience and sought PIM remedies (e.g., used galactagogues, increased diet quantity and quality) and whose families helped manage breastfeeding continuation and working. Previous studies also found that other urban mothers gave up breastfeeding when faced with obstacles such as child biting [32, 36] and breastfeeding refusal [36]. The results implied that mothers could not breastfeed because they did not have the skills to solve breastfeeding difficulties [32]. As recognised by other works in Indonesia [35] and Singapore [44], maternal self-efficacy was the critical factor of PIM. Enhancing breastfeeding self-efficacy may reduce PIM, thus extending the breastfeeding duration [45]. Healthcare providers should combine educational strategies with support to improve breastfeeding self-efficacy, such as verbal encouragement, vicarious experience through peer support groups, and involving husbands or families. Hence, capacity building for health workers in breastfeeding promotion is needed to better support breastfeeding practices.

#### Settings

##### *Family factors*

Family support contributed to mothers’ thoughts or feelings toward breastfeeding, leading to continued breastfeeding intention and practice [46]. Following earlier research [43], families can support mothers to continue breastfeeding in several ways, including offering emotional support (e.g., mothers supported to continue to breastfeed for at least two years) and instrumental support (e.g., offering support with childcare while they worked). Conversely, a lack of family support may discourage breastfeeding [47]. For example, family members may suggest to mothers or approve maternal decisions to terminate breastfeeding and use formula feeding. Providing family members (e.g., fathers, grandmothers) with knowledge and breastfeeding management skills is vital to enhance their support towards breastfeeding, especially when mothers encounter breastfeeding obstacles. Therefore, health practitioners should reach out to lactating mothers and their families during breastfeeding promotion programmes.

##### *Healthcare services*

Rural mothers’ compliance with breastfeeding recommendations was high. The rural midwives and village *kader* particularly emphasised breastfeeding’s health and financial benefits over formula milk. Rural mothers and their families accepted the midwife’s advice because they highly respected her and the suggestions were compatible with their conditions. They mostly entrusted the decision to health practitioners, including breastfeeding decision-making. Such dependency on health-related decision-making was higher among people with lower education [48]. Therefore, healthcare providers and policymakers should understand the socioeconomic and sociocultural factors behind maternal breastfeeding practices when providing breastfeeding advice and policies.

Most health workers supported breastfeeding for up to two years. According to the findings, most mothers, particularly those living in urban areas, agreed that breastfeeding should continue until their children aged two years old. While international consensus recommends breastfeeding for two years or longer, the Indonesian Ministry of Health regulations [49, 50] and various national guidelines, including *posyandu* handbook [19], only suggest breastfeeding for up to two years. As a result, most of our participants deemed the age of two years to be the upper limit for breastfeeding continuation because of the guidelines’ wording. Only in 2020 did the Indonesian Ministry of Health’s guidance on infant and young child feeding begin to urge that breastfeeding be continued until the children are two years old or older [51]. Indonesian policymakers and public health programmers should be aware of the differences in these recommendations. Therefore, revisiting and revising breastfeeding-related regulations and guidance are key for promoting breastfeeding continuation for up to two years or beyond at all levels.

##### *Employment factors*

Unemployed mothers dominated rural families. As housewives, they had more flexibility to take care of their children, including breastfeeding, than employed mothers. The results follow previous studies [13, 44, 52]. In contrast, one in three urban mothers were working outside the house. Women’s employment influenced the decision to cease breastfeeding [32, 33, 36, 44]. With three months of maternity leave [53], our urban participants found it challenging to sustain breastfeeding. First, not all mothers had sufficient support and facilities for expressing, storing, and preparing breast milk. Second, although breast pumping could be a solution to maintain breastfeeding while working, some mothers recognised the difference in milk production between direct breastfeeding and milk expression via pumps. Third, mothers felt that expressing breast milk was stressful. Likewise, earlier publications reported that breastfeeding for women in employment was generally demanding [52, 54, 55]. Breastfeeding is an adaptive process, depending on maternal experience and circumstances. Instead of emphasising the strict breastfeeding recommendations, healthcare professionals should enhance maternal awareness and offer mothers learning opportunities without adding stress related to the pressure to follow guidance [3]. Since employed mothers encounter more varied breastfeeding issues, they need adequate support from families, workplaces, health providers, and governments to improve breastfeeding outcomes. For example, families can help prepare stored breast milk [43]. Employers can provide facilities for expressing and storing breast milk and make work arrangements more flexible [42]. Health workers should give more practical advice about breastfeeding management for employed mothers and their families [56]. Finally, governments can offer programmes to supply breast pumping tools and provide adequate paid maternity leave for mothers in employment [23].

### Strengths and limitations

We recruited mothers of young children aged 6–23 months to capture breastfeeding continuation practices recommended by the WHO for at least two years. We compared lactating mothers’ experiences in rural and urban settings to better understand the topic. However, despite data saturation, a difference in participant numbers between rural and urban settings might not equally represent the findings. We conducted a *posyandu*-based recruitment process because *posyandu* is the closest health facility to society and is ubiquitous across Indonesia. Nevertheless, this procedure might limit our study to include mothers from higher-income families.

### Policy implications

This study identifies factors influencing breastfeeding sustainability at individual and setting levels, which are linked to structural factors. At the individual level, perceived low milk supply, child hunger and satiety, and formula milk benefits have led to breastfeeding termination and formula-feeding practices. Formula milk companies often use these challenges to promote their products and position them as equivalent or superior to breast milk [39]. In Indonesia, the marketing of breast milk substitutes is regulated under the Law of the Republic of Indonesia Number 33 in 2012 [49]. However, the law exclusively addresses marketing for the first six months and only within the health system, not at the retail level where the majority of marketing takes place [57]. National law should fully legislate the International Code of Marketing of Breastmilk Substitutes [23]. Such legislation should curtail formula milk marketing activities and be accompanied by effective monitoring and enforcement that are independent of commercial influence [23, 39, 58]. While the implementation of this law remains unclear due to a lack of data [23], there is an urgent need to assess the novel challenges of digital marketing of breast milk substitutes [39, 59].

Our findings at the setting level suggest maternal employment as one of the primary causes of not breastfeeding or providing formula milk. The Government of Indonesia has regulated maternity leave for 1.5 months before and 1.5 months after delivery for employed women [53], which is below the WHO’s standard of exclusive breastfeeding (26 weeks) and the International Labour Organization’s standard of maternity leave (14 weeks) [23]. While Indonesia’s law on breastfeeding is spread out among multiple documents [60], none has addressed paid maternity leave and the duration of nursing breaks at the workplace. The provision of breastfeeding facilities is carried out following the organization’s capabilities, indicating high dependency on the organization’s conditions [61]. Thus, there is a need for comprehensive policy support on maternity rights protection at the workplace, including extended duration of paid maternity leave [23] and appropriate breastfeeding facilities and breaks [23, 42].

## Conclusion

This is the first Indonesian study to compare breastfeeding continuation beyond six months between rural and urban women. Rural mothers practised continued breastfeeding for a longer duration than urban mothers. On the other hand, formula feeding practice has become standard in urban settings. Individual, family, community, healthcare, and employment factors determined breastfeeding continuation practices up to two years of age. Therefore, breastfeeding promotion programs should integrate educational interventions with a robust support system from families, communities, health workers, and workplaces. From the prenatal period, healthcare practitioners should equip mothers with adequate breastfeeding information, motivation, and practical skills, including overcoming breastfeeding barriers. Simultaneously, such interventions should engage husbands, families, peers, and local community leaders and consider the sociocultural context. Furthermore, regular training for healthcare workers and the establishment of peer support groups will help mothers access quality breastfeeding education. Our findings are important for researchers and public health workers to design community-based breastfeeding interventions tailored to different community contexts. Finally, policymakers should oversee the implementation and enforcement of breastfeeding policies to protect mother and child rights to breastfeeding from the formula milk industry’s influence and to promote and support breastfeeding in households, work environments, and healthcare systems.

## Data Availability

The datasets used and/or analysed during the current study are available from the corresponding author upon reasonable request.

## List of abbreviations

PIM: Perceived insufficient milk
UNICEF: United Nations Children’s Fund
WHO: World Health Organization
